# Famous Poisoners in Fiction

**Published:** 1893-10-07

**Authors:** 


### Famous Poisoners in Fiction.

### VII.—THE CHILD OF CRIME.

Wilkie Collins was always especially noted for his plots, but this did not prevent his characters from being lifelike. "Jezebel's Daughter" is one of his lesser known works, but it is as clever in its way as is the "Moonstone," the "Woman in White," and "Poor Miss Finch." Nevertheless, there is something repellant in the book. Last week's article was all about Madame de Villefort, Dumas' marvellously drawn representation of a perverted mother's love, and to-day we deal with the same subject. Madame Fontaine could, she tells the hero, have borne poverty and disgrace for herself, but not for her child;' therefore the wife of the great scientist converts the poison her husband had intended to use for the benefit of mankind into an instrument of death against the woman who sternly refuses to shield her child's name from shame and dishonour.

There is one great difference between the characters of Madame de Villefort and Madame Fontaine—the former sins with a calm deliberation, and not because she is urged on by the tyrant Necessity, but simply from pure love of gain, but the latter sins in desperation. "For Minna's sake, for Minna's sake." This is the excuse Madame Fontaine gives to her own heart for the crime she meditates. The deed done, she is filled with horror, with shame, for she is no brazen-faced vixen who rejoices in her act of vengeance. Her end, too, satisfies poetic justice, she dies by means of her own poisons; this child of crime finds that crimes like curses come home to roost; whilst Mrs. Wagner proves, through her life being saved by her devoted attendant, that the bread of kindness cast upon the waters of Time comes back again "after many days" with a threefold blessing.

The celebrated doctor is supposed to have sought to re-discover the poisons of the fifteenth and sixteenth centuries, and to have partially succeeded. One of these poisons he is supposed to have called "Alexander's Wine," another "The Looking Glass Drops." As no definite description is given of these poisons, it is almost impossible to say whether Wilkie Collins wrote of real poisons, or of some which had no being save in the recesses of his fertile brain; there is little doubt, however, that the antidote named, and which administered by Jack Straw saved his mistress' life, was the wine mandragora. This wine, Atrope Mandragora (to give its full name), was made from a plant which grows in Greece, a species of the English "Deadly Nightshade." It is never used now, but both as a poison and a medicine it was known to the medicos of the Middle Ages, and was discovered apparently in far more ancient days, for both Dioscorides and Pliny the Younger mention it in their writings. Dryden also sings of the "Mandrake dread." It seems to have been used by the physicians of Greece and Rome for the prevention of pain during operations, after the same manner as chloroform is now given, and it is said that when the patient was under its influence the greatest agony from knife or torture was unfelt. Many believe that this was the drink offered to Christ, and of which He refused to take, because that

> He would bear all that He might pity all,
> And rather would He wrestle with deep pain,
> Than cloud His soul so strong in agony.

It is a known fact that this wine was often administered to those suffering the tortures of crucifixion, and this is the reason why the order was so often given that the legs of the crucified should be broken, for otherwise the death-like trance produced was many a time taken for death, the victims afterwards reviving. It was probably because some such theory had arisen regarding the Lord's resurrection that the fourth Evangelist insisted so emphatically upon the piercing of the side of the dead Jesus by the Roman soldier.

The effect of this wine was to throw the sufferer into a death-like swoon, so exactly resembling death itself that all but the greatest experts would be deceived by it, and take the sham for the reality.

Not only our great dramatist, but novelists of every land and century have made use of this convenient plant for saving their victims' endangered lives. Valentine, in "Monte Christo," is thus preserved from her step-mother's undying hatred, and in this book, "Jezebel's Daughter," Mrs. Wagner is by its use uplifted, as it were, from the very grave itself to find herself lying in the dead-house of the great German city. The account of the apparently dead woman drawing back, with marble fingers, the heavy curtains, and appearing suddenly before the tipsy night watcher and his companions, is thrilling in the extreme. But the whole story verges too much on the brink of improbability to be exactly interesting reading. It is a strange fact, interesting to those who know aught about morphia maniacs, that in olden times many persons desiring oblivion for awhile from life's cares and troubles used to take doses of the wine Mandragora; they were called "mandragorates," after their favourite drug. The length of the trance produced was in accordance with the strength of the dose taken, and during the period of recovery they used to give vent to a series of fearful shrieks. Thus is the foundation of the popular superstition, still prevalent in the West country, that the mandrake root shrieks when drawn from the ground. Juliet, in "Romeo and Juliet," refers to this old world belief in the line:

> And shrieks like mandrakes torn out of the earth.

The drug is still used for medical purposes, but it has been greatly superseded by newer discoveries, and holds not now the honourable place it once held in that sanctum sanctorum—the scientist's medicine chest.

